# Recent Advances in Porous Polymers for Solid-State Rechargeable Lithium Batteries

**DOI:** 10.3390/polym14224804

**Published:** 2022-11-08

**Authors:** Junyan Zou, Teng Ben

**Affiliations:** 1 Zhejiang Engineering Laboratory for Green Syntheses and Applications of Fluorine-Containing Specialty Chemicals, Institute of Advanced Fluorine-Containing Materials, Zhejiang Normal University, Jinhua 321004, China; 2 Key Laboratory of the Ministry of Education for Advanced Catalysis Materials, Institute of Physical Chemistry, Zhejiang Normal University, Jinhua 321004, China; 3 Guangzhou Key Laboratory of Vacuum Coating Technologies and New Energy Materials, Department of Physics, Jinan University, Guangzhou 510632, China

**Keywords:** solid-state, rechargeable lithium batteries, polymers, porous polymers

## Abstract

The application of rechargeable lithium batteries involves all aspects of our daily life, such as new energy vehicles, computers, watches and other electronic mobile devices, so it is becoming more and more important in contemporary society. However, commercial liquid rechargeable lithium batteries have safety hazards such as leakage or explosion, all-solid-state lithium rechargeable lithium batteries will become the best alternatives. But the biggest challenge we face at present is the large solid-solid interface contact resistance between the solid electrolyte and the electrode as well as the low ionic conductivity of the solid electrolyte. Due to the large relative molecular mass, polymers usually exhibit solid or gel state with good mechanical strength. The intermolecules are connected by covalent bonds, so that the chemical and physical stability, corrosion resistance, high temperature resistance and fire resistance are good. Many researchers have found that polymers play an important role in improving the performance of all-solid-state lithium rechargeable batteries. This review mainly describes the application of polymers in the fields of electrodes, electrolytes, electrolyte-electrode contact interfaces, and electrode binders in all-solid-state lithium rechargeable batteries, and how to improve battery performance. This review mainly introduces the recent applications of polymers in solid-state lithium battery electrodes, electrolytes, electrode binders, etc., and describes the performance of emerging porous polymer materials and materials based on traditional polymers in solid-state lithium batteries. The comparative analysis shows the application advantages and disadvantages of the emerging porous polymer materials in this field which provides valuable reference information for further development.

## 1. Introduction

With the improvement of living standards, portable electronic products are becoming more and more popular, and rechargeable lithium batteries have become an indispensable part of our life. However, traditional commercial lithium batteries have potential safety hazards such as liquid electrolyte leakage, flammability [1,2] or fire and explosion caused by a large amount of heat-in-process use of the battery [3]. Although the above-mentioned hidden dangers can be effectively controlled through cooling schemes [3,4], solid-state rechargeable Lithium Batteries are considered to be the best candidate to fundamentally cope with the challenge, because their electrolytes do not contain any flammable liquids, and the rigidity of solid materials effectively resists the formation of lithium dendrites to avoid short circuits [5,6,7,8,9]. However, the further development of all-solid-state rechargeable lithium batteries still needs to break through the difficulties of how to reduce the interface resistance between the electrolyte and the electrode, as well as how to improve the ionic conductivity and stability of the electrolyte [10,11]. To this end, researchers have made many attempts to improve the performance of all-solid-state lithium batteries since the initial exploration of electrolyte materials. Some combine the advantages of organic materials [12,13,14] such as polymer electrolytes and inorganic materials [15,16,17] such as ceramic electrolytes to prepare hybrid electrolytes to improve ionic conductivity [18,19,20,21]; some add functional additives to electrodes and electrolytes to increase interfacial contact [22,23]; Some insert new coatings between electrolytes to reduce interfacial resistance [24,25,26], etc. Most of these attempts are inseparable from the participation of polymers, which play a significant role in the improvement of battery performance.

As we all know, polymer materials are formed by repeated connection of many identical and simple structural units. Due to the existence of covalent bonds between molecules, the physical and chemical properties of polymers are relatively stable. Moreover, by changing the types of monomer molecules or molecular binding, it can make the target polymer satisfy specific performance such as flexibility, stretching, rigidity, toughness, film formation, and high temperature resistance which is the special requirements for electrodes and electrolyte materials of solid-state Lithium batteries. Therefore, polymers are considered to be advantageous in the application of all-solid-state rechargeable lithium batteries.

The traditional polymers used in solid-state lithium batteries are linear polymers such as Polyethylene oxide (PEO), Poly (vinylidene fluoride) (PVDF), polymethyl methacrylate (PMMA), Polyacrylonitrile (PAN), etc. In the past decade, some porous organic polymers have emerged for solid-state lithium batteries, including amorphous porous aromatic frameworks PAFs and crystalline covalent organic frameworks COFs, metal organic frameworks MOFs, etc., with their high chemical absorption rate and ionic conductivity, it is expected to become the most popular materials for next-generation solid-state lithium chemical battery electrode materials, ionic conductors, interface stabilizers, and functional precursors.

**Figure 1:**
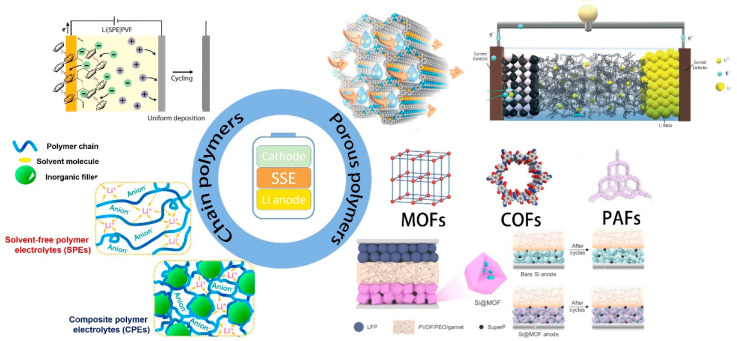
Applications and contributions of polymers in All−Solid−State Rechargeable Lithium Battery. Adapted with permission from ref. [27]. Copyright 2019 Elsevier. Ref. [28]. Copyright 2021 American Chemical Society. Ref. [29]. Copyright 2019. John Wiley and Sons. Ref. [30]. Copyright 2022 American Chemical Society.

The application of porous polymers in solid-state Lithium Batteries has made some revolutionary progress. However, as research is still in its infancy, relevant scholars have discussed different perspectives. This review reveals its advantages and deficiencies through comparison of the performance of porous polymers and traditional polymers. It clarifies the direction of porous polymers that still need to work hard and looks ahead to the application of porous polymers in solid-state batteries. The applications and contributions of polymers in All-Solid-State Rechargeable Lithium Battery are shown in Figure 1.

## 2. Challenges of All-Solid-State Rechargeable Lithium Batteries

In order to deal with the safety hazards such as electrolyte leakage, flammability, explosion [31,32,33] and the growth of lithium dendrites piercing the separator and causing short circuit in traditional lithium batteries, the solid-state lithium-ion conductive materials of All-Solid-State Lithium Batteries operate as the separator and electrolyte can eliminate the possibility of liquid electrolyte leakage, which improves the safety of rechargeable lithium batteries [34,35]. In addition, solid-state batteries have longer cycle life than traditional lithium batteries due to their slow side reactions compared to liquids. Therefore, compared to the continuous optimization of traditional lithium-ion batteries, it is undoubtedly a better choice to explore the core components of batteries to develop the next generation of batteries represented by all-solid-state rechargeable lithium batteries.

At present, all-solid-state electrolytes are mainly divided into three categories: solid-state inorganic electrolytes, solid-state polymer composite solid-state electrolysis and solid-state polymer electrolytes [36,37]. Solid-state inorganic electrolytes such as oxide electrolytes, sulfide electrolytes and halide electrolytes have their own strengths in mechanical strength, ionic conductivity, and low grain boundary resistance, but high preparation costs, complicated preparation procedures, poor compatibility with cathode materials, as well as water and oxygen instability need to be broken through [38]. Solid-state polymer electrolytes meet the current development requirements due to their good flexibility, low preparation cost, and good compatibility with electrodes, but the ionic conductivity is generally lower than that of inorganic solid-state electrolytes, especially at room temperature. Combining the advantages of the two electrolytes, the organic-inorganic polymer composite electrolyte thus becomes the third candidate material for the preparation of all-solid-state batteries.

Based on the above discussion, polymers and polymer-based solid electrolytes are more suitable for use in all-solid-state rechargeable lithium batteries. Despite the advantages of solid-state lithium batteries in terms of safety, cycle life, and preparation procedures, the solid-state rechargeable Lithium batteries still face some challenges: (1) Recent studies have found that lithium dendrites can still pierce the seemingly rigid solid-state electrolyte due to uneven deposition of lithium metal, causing battery short-circuits; (2) The solid-solid contact between the electrolyte and the electrode is affected by the stress generated by the electrochemical cycle, which leads to the increase of the contact resistance and the decrease of the battery performance [39]; (3) What is more noteworthy is that the lithium ion conductivity of many solid-state electrolytes has not made a great breakthrough at present [40]. Polymers play an important role in the electrolyte, electrode, and interface stability of all-solid-state rechargeable lithium batteries. In order to improve battery performance, many studies have been carried out on the effects of polymers on all-solid-state rechargeable lithium batteries.

## 3. Solid-State Polymer Electrolytes

In all-solid-state rechargeable lithium batteries, the solid-state electrolyte is located between the cathode and the anode, acting as an electrolyte and a separator, so the performance of the solid-state electrolyte is crucial to the performance of the entire battery. As an all-solid-state polymer electrolyte for lithium batteries, it should meet the requirements of battery applications, such as high ionic conductivity, high lithium-ion migration number, excellent mechanical properties to resist the puncture of lithium dendrites, good chemical and thermal properties stability, etc. [27,41,42,43].

In order to meet the performance requirements of the above-mentioned for solid-state lithium batteries, since polymers were formally proposed to use in lithium-ion batteries in 1970s [44], domestic and international researchers have been focusing on the application of polymers and polymer-based composite electrolytes in All-Solid-State lithium-ion batteries, including the exploration of ion transport mechanisms and the development of new polymer electrolyte systems [45,46].

In this section, we first introduce polymer matrix and polymer-based composite electrolytes, and then describe some current research work on the performance impact of all-solid-state rechargeable lithium batteries, and propose possible future development directions.

### 3.1. Polymer Matrix

The polymer matrix can be divided into polyethylene oxide (PEO) [47], polyacrylonitrile (PAN) [48], polyvinylidene fluoride (PVDF) [49], polymethyl methacrylate (PMMA) [50] and other polymers (PVC, PA, PCA) [51,52,53] according to the type of main chain. The structure, properties and applications of the polymer matrix are summarized in Table 1.

#### 3.1.1. PEO

Polyethylene oxide (PEO) is the polymer with the strongest complexing ability and the most widely used polymer electrolyte matrix [27,43]. The flexible polyether segment of PEO molecule can be complexed with alkali metal ions (Li^+^). Under the action of the electric field, Li^+^ continuously undergoes “coordination-dissociation” movement with the ether oxygen atom along with the thermal movement of the molecule, achieving directional and rapid migration. The structure and transport process are summarized in Table 1. However, due to the high crystallinity of PEO, its ionic conductivity is poor [54]. Therefore, PEO is usually blended, copolymerized, and cross-linked with other polymers, or plasticized by adding low-molecular-weight solvents, adding nanoparticles, etc. to prepare a composite electrolyte to improve its performance.

#### 3.1.2. PMMA

In addition to the above drawbacks, it is also worth noting that the low dielectric constant of PEO cannot completely separate the ions dissolved in it, and it is easy to cause ion aggregation to affect the migration of Li^+^ [43]. Introducing strong polar groups into the polymer molecular structure can improve the dielectric constant [55,56], so polymers with strong polar carbonate groups have attracted extensive attention of researchers, and polymethyl methacrylate (PMMA) is the most widely used representative [57,58].

The research on the application of PMMA polymer matrix in all-solid-state lithium batteries first started in 1985 [59]. Due to the abundant raw materials, easy synthesis and low cost of PMMA, it has aroused the strong interest of researchers in PMMA polymer electrolytes. The research on the mechanism and modification methods has also made good achievements in recent years.

#### 3.1.3. PVDF

The PMMA-based polymer has a stable structure and is stable to metals, and the resistance of the passive film formed on the contact surface with the electrode is small. However, PMMA has the disadvantages of being hard and brittle after film formation, poor flexibility, and poor mechanical strength [45].

Poly (vinylidene fluoride) (PVDF) material has high melting point, good thermal stability, good electrochemical stability and good film-forming properties, and its high dielectric constant helps to promote the ionization of lithium salts, which can well make up for the shortcomings of PMMA and become a kind of Emerging polymer electrolyte matrices [60]. The ionic conduction mechanism of PEO can be expressed as: Li^+^ is complexed with the coordinating groups on the polymer chain after the dissociation of the lithium salt, and transported in the system along with the molecular thermal motion [61]. Unlike PEO polymers, PVDF is a non-coordination polymer, Li^+^ has not direct interaction with the polymer main chain, instead, after the dissociation of the lithium salt, countless anions line up on both sides to form a channel, and the coordination mode of the freely moving Li^+^ and each anion is similar to that in the pure salt crystal structure. The coordination site jumps to the one that coordinates with the next anion, completing the migration, as shown in the schematic diagram of the ion transport mechanism in Table 1 [62].

However, the homopolymer structure of PVDF leads to a high intramolecular crystallinity (65–78%), which is not conducive to ionic conduction. Therefore, the modification methods for PVDF are usually to interact with other polymers or inorganic fillers to reduce the crystallinity and improve the electrolyte absorption rate, or to improve the ionic conductivity by cross-linking, blending, adding initiators [63,64,65,66,67].

#### 3.1.4. PAN

Polyacrylonitrile (PAN) is a polymer containing nitrile group (C≡N) with strong electron withdrawing ability [68]. The ion migration mechanism of PAN is similar to that of PEO. Li^+^ is complexed with the coordination group on the polymer chain after dissociation in the lithium salt, and the coordination site is migrated with the movement of the polymer matrix or jumps from a chain to another chain of the polymer [69]. But the difference is that the movement of Li^+^ along the PAN segment chain at room temperature does not affect the ionic conductivity very much, and the Li^+^ transfer number in PAN-based electrolyte is higher than that of PEO [70,71]. Its strong electrochemical stability, strong heat resistance, good flame retardancy and simple synthesis makes it become an ideal candidate for polymer electrolyte matrix materials [20]. The structure of PAN is shown in Table 1.

However, the research applied to solid-state lithium batteries found that the poor wettability of PAN and electrodes leads to high interface impedance of lithium batteries, and the performance of batteries is not ideal. Therefore, it is usually necessary to add plasticizers or inorganic fillers to PAN to improve ionic conductivity and interfacial compatibility [71,72,73,74].

**Table 1:** Summarized for the structure, properties and applications of the polymer matrix. Adapted with permission from ref. [75] Copyright 1995 Elsevier. Ref. [76] Copyright 2021 American Chemical Society Ref. [62] Copyright 2020 Elsevier. Ref. [77] Copyright 2019 Elsevier.

Polymer Matrix	Repeating Unit	Transport Process	References
PEO	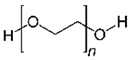	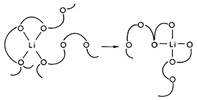	[75]
PAN	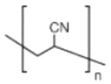	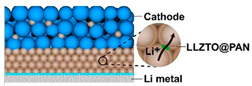	[76]
PVDF	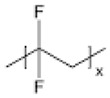	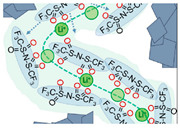	[62]
PMMA	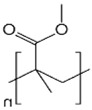	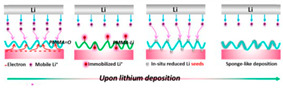	[77]

It can be seen that different polymer matrices have their own advantages, and they can complement their own defects through blending or copolymerization and improve the performance of solid-state lithium batteries from different aspects.

### 3.2. Solid-State Polymer Electrolytes

Based on the research background of polymer electrolytes and the defects of polymer electrolytes in the past, a series of new studies have been carried out in recent years to develop new polymer electrolytes to improve the performance of solid-state lithium rechargeable batteries, mainly including all-solid-state polymer electrolytes, polymer-inorganic composite electrolytes and porous polymer electrolytes.

#### 3.2.1. All-Solid-State Polymer Electrolyte

All-solid polymer electrolytes consist of two parts: a polymer matrix and a lithium salt that can be dissolved in it. At present, all-solid polymer electrolytes generally suffer from low room temperature ionic conductivity or high electrolyte interface impedance, which greatly restricts all-solid-state polymer electrolytes [78]. For the practical application of polymer electrolytes, researchers have modified and optimized the polymer structure, including copolymerization (blending) [79], cross-linking [80] or grafting [81], to prepare new all-solid-state polymer electrolytes in recent years.

Subir et al. investigated the effect of blending host polymers on solid polymer electrolyte (SPE) membranes doped with ammonium iodide (NH_4_I) salts. Experiments have shown that poly (ethylene oxide) (PEO)-poly (vinylidene fluoride) (PVDF) polymers mixed in suitable ratios can strengthen the amorphous part of the polymer matrix and facilitate fast ionic conduction through it. The addition of PVDF enhances the ion-polymer interaction in the system, resulting in more ion dissociation, while PEO provides a path for ion conduction. When the ratio of PEO and PVDF is 8:2, the ionic conductivity of the solid polymer electrolyte is up to 1.01 × 10^−3^ S/cm [82]. PEO can also be blended with other polymers such as polyvinylpyrrolidone (PVP) [83], poly (methyl methacrylate) (PMMA) [84], polystyrene (PS) [85], 2,4-toluene diisocyante/polyethylene glycol (TDI/PEG) [86], which can also significantly improve the conductivity of solid electrolytes. In addition to the most commonly used polymer matrix PEO, other emerging polymers can also be copolymerized or blended by the same method to prepare new solid polymer electrolytes. According to the different molecular structures of polymers, they can be divided into block copolymerization, graft copolymerization et al. [87,88,89]. The experimental results show that the performance of polymer solid electrolytes prepared by blending or copolymerization of single polymer electrolytes is significantly improved. It also significantly improves the cycle life and dendrite resistance of solid-state rechargeable lithium batteries.

Although the PEO polymer electrolyte can effectively improve the performance of the solid polymer electrolyte itself by means of blending, copolymerization, grafting, etc., the compatibility between the electrolyte and the electrode cannot be ignored in the application of solid lithium batteries. The unstable contacts of PEO and LiCoO_2_ etc., cathode materials hinder its potential application in high-energy-density batteries. Fu et al. designed an ultrathin double-salt PEO-based polymer electrolyte (DPPE) with a cross-linked network to address these issues [90]. The formed cross-linked network greatly increases the amorphous region and room temperature ionic conductivity of PEO-based polymer electrolyte. The cross-linked network effectively inhibits the decomposition of PEO segments when contact with cathode and effectively maintains the chemical stability between the electrode and the electrolyte. The cycle performance and capacity retention rate of the battery are both satisfactory. This is undoubtedly good news for PEO-based solid-state electrolytes to be used in high-voltage solid-state rechargeable lithium batteries at ambient temperature and Many PEO-based polymers refer to this strategy to modify polymer electrolytes [91,92,93]. Recently, Tsai et al. conducted an in-depth study on the relationship between solute diffusion and crosslink density, demonstrating that polymer crosslinking has a significant effect on regulating solute diffusivity and polymer mechanical properties [94].

#### 3.2.2. Polymer-Inorganic Composite Electrolytes

The low ionic conductivity and mechanical strength of polymer electrolytes seriously affect their practical application in solid-state rechargeable lithium batteries. As early as 1982, Weston and Steele found that adding 10% inorganic solid fillers to PEO-based polymer electrolytes, the mechanical properties of the electrolyte can be improved, but the effect on the ionic conductivity is small [95]. Since then, the research of a new electrolyte combining polymer electrolyte and inorganic solid electrolyte has started a new journey. The cations in the inorganic material Al_2_O_3_ can combine with the ether oxygen groups on the polymer segment as Lewis acid through Lewis acid-base interaction like Li^+^, inhibit the crystallization of the polymer, and form a physical cross-linked network system with the polymer segment, which can improve the mechanical properties and thermal stability of polymer electrolytes, but the problems of ionic conductivity and interfacial stability have not been solved. Such inorganic materials do not have ionic conductivity, so they are also called inert fillers. MgO, TiO_2_, SiO_2_ and ZnO also belong to such inert inorganic fillers.

Another inorganic filler is active inorganic filler. Unlike inert inorganic fillers, its conductivity is much higher than that of polymer matrix and lithium ions can migrate in fillers. Therefore, it can effectively improve the ionic conductivity of composite electrolytes to a certain extent. The garnet-type solid electrolyte is the active inorganic filler with the highest conductivity [96]. Recently, He et al. studied a garnet-type inorganic-polymer composite solid-state electrolyte Li_6.4_La_3_Zr_1.4_Nb_0.6_O_12_-PEO, with a high conductivity of 1.4 × 10^−3^ S/cm at 60 °C and an electrochemical window of 5.2 V, the solid-state battery prepared with this electrolyte can be charged and discharged normally at room temperature and has a high-capacity retention rate after cycling. Other similar composite polymer electrolyte experimental data show that its conductivity is better than that of inert inorganic filler composite solid electrolyte, and the battery interface resistance is greatly reduced, and the stability and capacity retention rate are greatly improved [33,97,98,99,100,101]. The high stability of the battery is due to the close surface contact between the polymer matrix and the electrode, which reduces the volume and increase the interface contact. Polymers play an important role [102].

These active inorganic filler-polymer composite electrolytes can also choose perovskite [103,104,105], NASICON [106,107,108], sulfide [109,110,111], etc. as the active inorganic filler part, and PVDF as the polymer matrix. The experimental results show that, composite electrolyte can not only retain the high ionic conductivity and mechanical strength of inorganic solid electrolytes, but also exert the advantages of high flexibility and high interfacial compatibility of polymer solid electrolytes [10,112]. Polymer-inorganic composite electrolytes have good ionic conductivity, cycling performance, and thermal/mechanical properties, and will always be a research hotspot.

#### 3.2.3. Porous Polymer Electrolyte

All-solid-state polymer electrolytes and polymer-inorganic composite electrolytes have achieved good results in improving ionic conductivity [113,114,115,116] and stabilizing the interfacial contact between electrodes and electrolytes [117,118,119] through various efforts in the past few decades. However, due to the increasing demand for high power, high voltage, long battery cycle life, etc. in practical applications, substantial improvements in their performance are still urgently needed.

In recent years, in order to explore solid electrolyte materials with better comprehensive properties, researchers have made many attempts. Among them, porous materials with natural internal pores have many excellent characteristics such as small specific gravity, large specific surface area, and strong adsorption performance. The inclusion of lithium-ion conductive materials or integrated functional groups in the pores is conducive to the transport of lithium ions in the pores, and the spatial network structure is mostly formed by covalent bonding, which has good thermal and chemical stability. This porous material with both function and structure can be widely used in electrochemical and energy storage devices, so it is considered as the best candidate for all-solid-state lithium battery electrolyte, and has received extensive attention and research in recent years. The most common porous polymer electrolytes are Metal Organic Frameworks (MOFs), Covalent Organic Framework (COFs), and the emerging Porous Aromatic Framework (PAFs).

**Figure 2:**
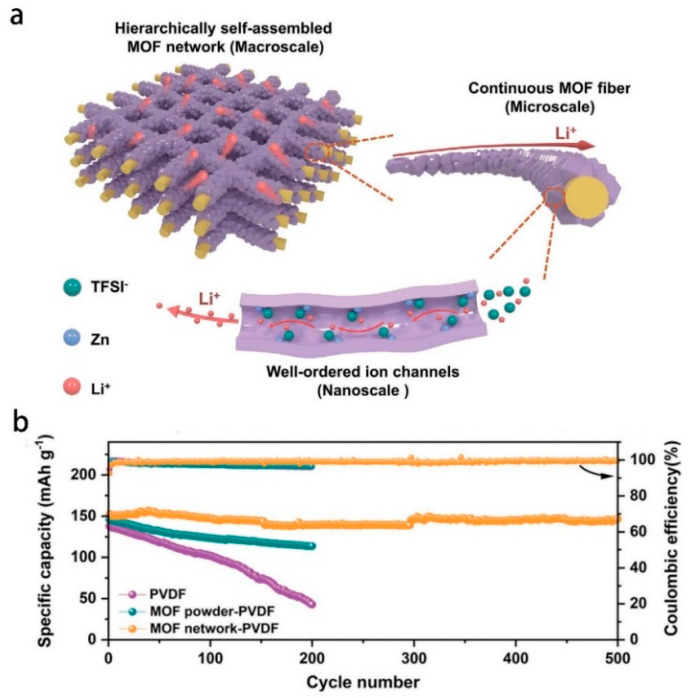
(**a**) Schematic illustration of hierarchically self−assembled MOF network as 3D ion conductor with continuous Li^+^ transport. (**b**) Cycling performances with different solid electrolytes. Adapted with permission from ref. [125]. Copyright 2022 John Wiley and Sons.

MOFs are crystalline porous materials with periodic network structure formed by inorganic metal centers (metal ions or metal clusters) and bridged organic ligands connected to each other through self-assembly, also known as coordination polymers [120,121]. It has both the rigidity of inorganic materials and the flexibility of organic materials, and has the advantages of high porosity, low density, large specific surface area, regular pore channels, adjustable pore channels, and diverse and tailorable topology [122]. Its development potential in lithium battery electrolyte applications was first reported by Long et al. in 2011 [123]. Since then, in-depth research has been carried out on porous polymer electrolytes including MOF-incorporated polymer hybrids, lithium salt-supported MOF hybrids, and pure MOFs [124]. Continuous porous structure of MOFs providing unimpeded continuous channels for lithium-ion transport, overcoming the obstacles of traditional composite electrolytes and polymer electrolytes, and effectively improving the performance of solid-state electrolytes. Recently, it was found that the movement of larger anions in MOFs can be selectively restricted by modifying the pore size of the MOFs, which acts like an ion sieve to clear the barrier for the transport channel of Li^+^ (Figure 2), thereby improving the ionic conductivity of the electrolyte and the performance of the battery [125]. Similarly, taking above advantage of the MOFs, adding them to conventional polymer electrolytes (PEO/LiTFSI) which significantly improves the ionic conductivity of electrolyte at room temperature [126]. The solid-state polymer electrolyte prepared by the addition of MOF not only improves the ionic conductivity, the stability of the electrochemical window and the lithium-ion migration number [127,128,129], but also effectively resists the piercing of lithium dendrites [130]. MOFs are considered as a promising new electrolyte for all-solid-state batteries. We summarized the recent studies about the performance of MOF-based solid-state electrolytes (SSEs) in Table 2.

The metal ions in the coordination center of MOFs materials and organic ligands are combined to form a three-dimensional network structure through coordination bonds, resulting in the disadvantage of poor thermal stability of most MOFs materials. Therefore, the ionic conduction of the electrolyte at high temperature is limited. UiO-66 and ZIF series MOFs materials have relatively good chemical stability and thermal stability, and UiO-66 and ZIF series MOFs materials have application prospects in mid-temperature ion conduction.

**Figure 3:**
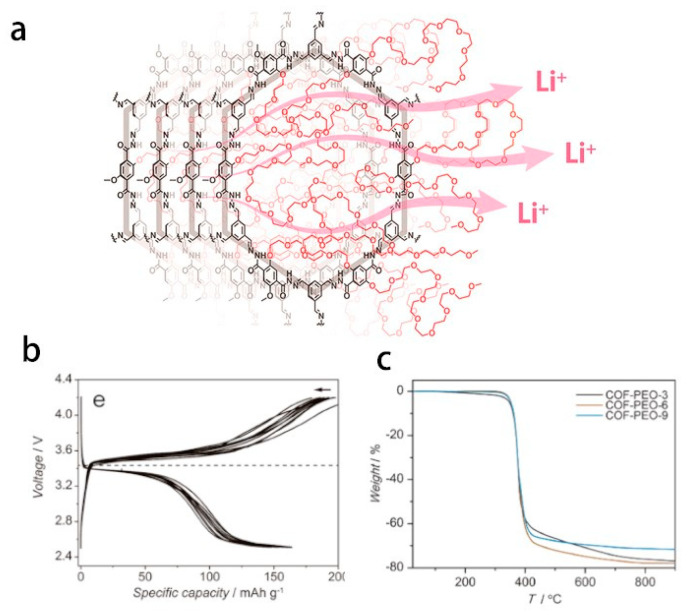
(**a**) Schematic diagram of fast directional transport of lithium ions in 2D COF structures by solvated Li^+^−polyethylene oxide (PEO). (**b**) Charge−discharge curves of an all−solid−state battery of LiFePO_4_/COF−PEO−9-Li/Li cell at a current intensity of 3.0 mAg^−1^ and cutoff voltage of 2.5 to 4.2 V at 100 °C. (**c**) TGA profiles measured at 10 °C min^−1^. Adapted with permission from ref. [140]. Copyright 2019 American Chemical Society.

Covalent organic frameworks (COFs) are crystalline porous materials with two-dimensional or three-dimensional periodic structures formed by covalently linking organic molecular units. With the advantages of low frameworks density, high porosity, and open pore structure, it is beneficial to ion conduction. Their applications in solid-state lithium battery electrolytes are receiving increasing research interest [139]. As shown in Figure 3, like metal-organic frameworks (MOFs), organic covalent frameworks (COFs) are porous crystalline materials that can precisely arrange organic building blocks to form an ordered structure, in which lithium ions are directionally transported in its ordered pores. But the porous polymer solid electrolyte remains structurally stable at high temperature (as high as 400 °C) [140], because the covalent network rigid structure of COFs endows them with better mechanical properties and thermal stability than MOFs. Recent research on COFs-based porous polymer electrolytes has made breakthroughs based on previous studies. The emergence of COFs-based porous polymer electrolytes opens a new chapter for the development of high-temperature solid-state lithium battery applications [28,141]. We summarized the recent studies about the performance of COF-based solid-state electrolytes (SSEs) in Table 3.

The regular and uniform pore structure of COFs is conducive to the transport of guest molecules in the pore, and the organic building units are connected by covalent bonds, which have high thermal stability and achieve rapid lithium-ion conduction above 100 °C. The pore size, crystallinity and other properties of COF materials depend on the building monomers, and the diversity of monomers endows COFs with diverse structures. If the pore size of COF is small enough, the more significant its ability to act as a solid electrolyte against lithium dendrites, because the solid-state electrolyte layer is dense enough. Therefore, the emergence of COFs has opened a new chapter in the study of solid-state electrolytes. However, the research and development of new COFs materials requires the design of new functional monomers and the study of new connection methods, which are very challenging work and require more efforts in the future.

**Figure 4:**
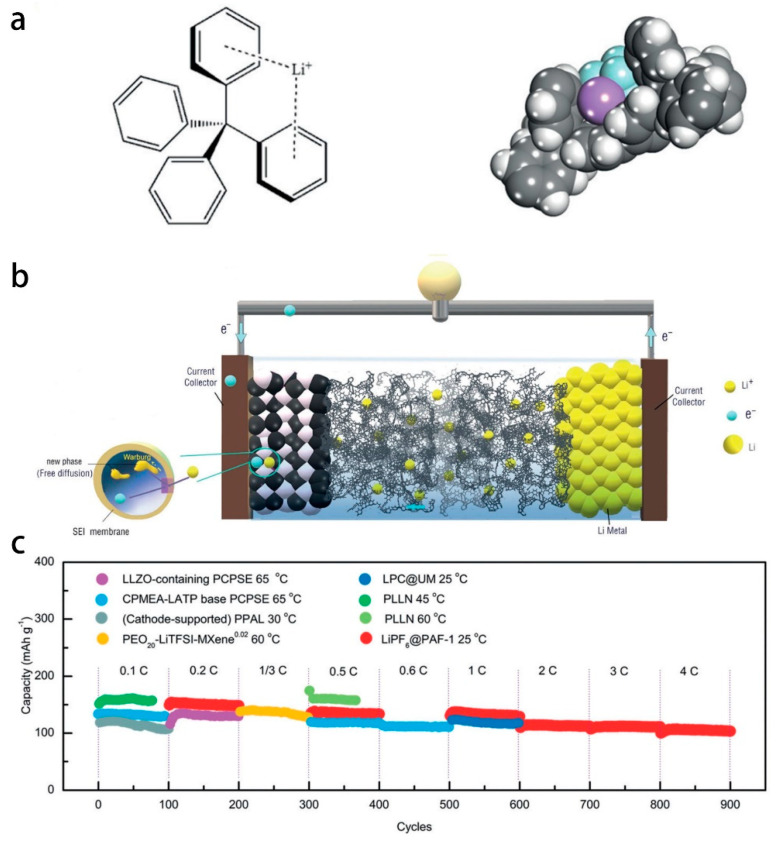
(**a**) Schematic showing the interaction of the Li^+^ ion with the faces of the two phenyl rings of the tetra phenyl methane nodes of PAF−1 and the van der Waals radius of each respective atom, Li ions are colored purple while F, P, C, and H atoms are colored light blue, pink, grey and white, respectively. (**b**) Mechanism of operation of the LiFePO_4_//LiPF_6_@PAF-1//Li cell. (**c**) Cycling performance of Li/LiFePO_4_ cells with other SSEs and LiPF_6_@PAF−1 in long term cycles (first 100 cycles). Adapted with permission from ref. [29]. Copyright 2019 John Wiley and Sons.

Porous Aromatic Framework (PAFs) are microporous network materials with open frameworks formed by linking aromatic units through covalent bonds. Its pores can be used as carriers for small molecular guests. The large specific surface area and excellent physical and chemical stability make such materials have broad application prospects in energy storage, adsorption, separation, and catalysis. In 2020, Ben’s research group first disclosed a major breakthrough in the application of PAFs in all-solid-state Lithium Battery electrolytes. The research group found that PAF-1 has high adsorption characteristics for LiPF_6_ due to the combination of the benzene ring in PAF-1 and the lithium ion in the lithium salt in the way of cation-π interaction. Lithium ion existed stably in the framework of PAF-1, while the continuous porous three-dimensional network and the large enough pore volume provided an unimpeded channel for the transmission of lithium ions and realizes the rapid operation of lithium ions. PAF-1 has high absorption rate and good stability of lithium salts, which meets the requirements of solid-state electrolyte lithium-ion batteries for high energy density, long cycle life and excellent rate performance. Compared with other solid-state electrolyte batteries with the same electrode materials, the battery can withstand higher current densities and longer cycle times in long-term cycling experiments, which shown in Figure 4 [29].

The application research of PAFs in solid-state lithium battery electrolytes is still in its infancy, and there are still many challenges such as the mechanical strength of the electrolyte, the research cost, and the adsorption performance of other lithium-ion conductive materials that need to be solved urgently.

### 3.3. Summary

Traditional linear polymers have low preparation cost, good solubility of lithium salts, and are easy to synthesize, but their low ionic conductivity at room temperature often requires composite preparation with other types of electrolytes to reduce their crystallinity. There is no chemical bond between the molecules of linear polymers, and the molecules can move relative to each other after being heated or stressed, so they are flexible and suitable for the preparation of gel electrolytes, but the mechanical properties are not good and the stability needs to be improved.

Porous polymers are porous network materials composed of building units connected by covalent bonds. It has the characteristics of high surface area, high adsorption performance, low density, adjustable pores, designable composition and structure, and easy modification. Compared with conventional linear polymer electrolytes, porous polymer electrolytes exhibit better mechanical properties and faster Li-ion transport due to the high chemical stability conferred by the presence of covalent bonds, and the robust framework can withstand higher voltages and currents density. However, the preparation is cumbersome and the cost is high. If a simple and inexpensive preparation can be realized, the development of porous polymer electrolytes will be better promoted.

## 4. Polymer Electrode Materials

The specific capacity of solid-state rechargeable lithium batteries depends on the cathode material, so the performance of the cathode material is crucial [149]. The traditional electrode materials for lithium batteries are mainly transition metal oxides and phosphates such as LiFePO_4_, LiCoO_2_, LiMn_2_O_4_, etc., but the problems of high cost, low stability and easy repeated expansion and contraction of volume are difficult to meet the needs of current use [150]. The improvement of energy density and power density is the top priority of current development. Polymer materials are rich in resources, designable structure, high theoretical capacity, good rate performance, and mechanical flexibility, and are considered to be an excellent choice for electrode materials [151,152,153,154,155,156,157].

### 4.1. Composite Polymer Electrode Materials

Different from traditional liquid electrolytes, the migration of anions in the solid electrolyte system is limited, and the insufficient solid-solid contact between the electrode and the electrolyte exacerbates the ion conduction, resulting in a sharp decrease in the battery cycle life, thus enhancing the adhesion between electrodes and electrolytes is the key to improve the performance of solid-state Lithium batteries. As early as 1995, Tsutsumi et al. used polyaniline-poly[p-styrenesulfonic acid-co-methoxy-oligo(ethylene glycol) acrylate] (PANI-PSSA-co-MOEGA) as a composite electrodes for all-solid-state rechargeable lithium batteries. Since the polymer electrode has anchoring anion sites, polyaniline and polyethylene oxide side chains can be doped, which serves as a significantly enhanced adhesion between the electrode and the polymer electrolyte. The prepared solid-state lithium battery exhibits good cycling performance and high capacity after 40 cycles [158]. Inspired by this, Ding et al. recently used the anion acceptor material polyvinyl ferrocene (PVF) as the cathode electrode to construct an advanced all-solid-state battery. The ferrocene unit on the polymer chain promotes Li^+^ and anion to work together as an effective charge carrier, the participation of anions in the electrode reaction weakens the effect of concentration polarization on the deterioration of the anode, and the ion utilization rate is significantly improved. The lithium metal battery prepared with the classical solid-state electrolyte (PEO-LiTFSI) exhibits excellent rate capability, and the results show that the discharge specific capacity reaches 107 mAh g^−1^ at a current density of 100 μA cm^−2^. It can be cycled stably for more than 4000 times at a current density of 300 μA cm^−2^ [159].

Polymers can also be used as electrode material additives to improve the performance of solid-state lithium batteries by enhancing the contact between electrodes and electrolytes. For example, the polymer binder can effectively alleviate the deterioration of the contact between the electrode materials caused by the volume change of the electrode materials during cycling. Hong et al. proposed a polytetrafluoroethylene (PTFE)-based ionomer with high Li-ion conductivity and good adhesion properties as a binder for electrodes. Its uniform distribution in the composite cathode facilitates the transport of Li^+^ and increases the interfacial contact between electrode materials. The battery prepared by the polymer composite cathode has good cycle stability and rate performance [22].

It is also worth noting that the electrolytes in solid-state lithium batteries, especially those based on the garnet type, have high stability to lithium metal, but the rigid contact with the solid electrode is not sufficient, leads to the volume change of the electrode during the charge-discharge cycle and causes the battery to fail. Li et al. reported a method of adding polymers as wetting agents to electrode materials to improve their compatibility with solid electrolytes. The study showed that an ion-conducting polymer electrode composed of PEG, LiTFSI and conductive carbon, assembled with LLZO electrolyte to form a solid-state rechargeable lithium battery. The charge-discharge cycle was up to 400 h without polarization [160]. With the rise of portable electronics, improving stability and cycle life is the future development trend of polymer electrode materials, and the next generation of lithium-ion batteries will be very promising because polymer electrode materials have natural advantages in these aspects [149].

### 4.2. Porours Polymer Electrode Material

In recent decades, researchers have found that some porous polymers, such as PIMs, COFs, MOFs, and PAFs, have inherent pores for ion transport without any ion-conducting groups. It can withstand higher current densities and have better chemical/electrochemical stability [161]. Some of these porous polymers have been used to study electrode materials for solid-state lithium batteries with good results.

**Figure 5:**
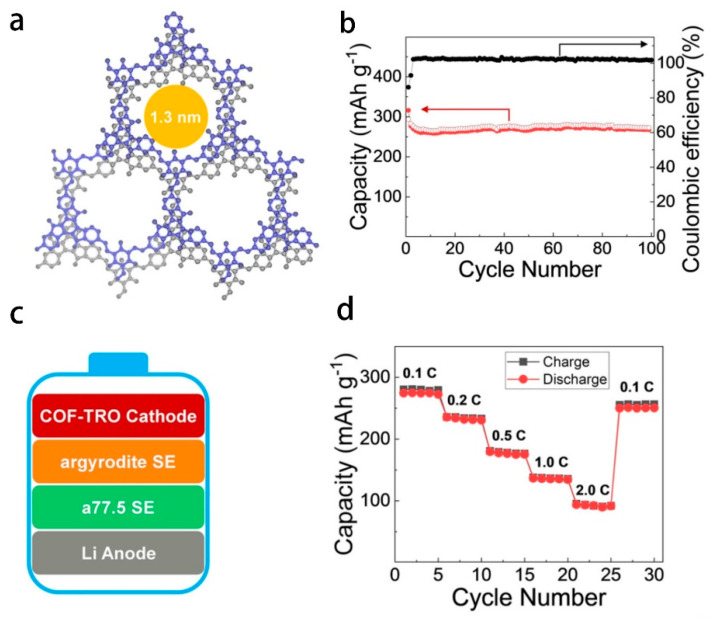
(**a**) Characterization of COF−TRO. (**b**) Cycling stability of COF−TRO at a current rate of 0.1 C and corresponding Coulombic efficiencies. (**c**) All−solid−state battery configuration. (**d**) Rate study of COF−TRO. Adapted with permission from ref. [162]. Copyright 2020 John Wiley and Sons.

Wang et al. reported for the first time that a novel covalent organic framework material COF-TRO was applied to the cathode active material of all-solid-state lithium batteries, showed in Figure 5. Benefiting from the characteristics of light weight and high crystallinity of covalent organic framework materials, a higher energy density can be obtained. The experiment found that the specific capacity of the all-solid-state lithium battery prepared by using COF-TRO as the cathode active material reached 268 mAh g^−1^, which is very close to the theoretical specific capacity calculated by the chemical structure. At the same time, the effective specific capacity hardly changed after more than 100 charge/discharge cycles, which also provides the possibility for the practical application of the material and provides a reference for the design of efficient all-solid-state lithium-ion battery materials in the future [162]. Due to their unique framework structure and the ability to accommodate a large number of lithium ions, COFs have been used in electrode materials with promising results [163,164].

Cathodes with high stability and strong ion transport properties can effectively deal with the challenges of low specific capacity and ion conductivity of solid-state lithium batteries, but the ability to suppress lithium dendrites is still a gap that solid-state lithium batteries need to cross. The high porosity of MOF effectively regulates uniform Li^+^ transport and deposition during charge and discharge, the strong framework has sufficient mechanical strength to inhibit the growth of Li dendrites and avoid low effective Li metal anode area capacity due to Li dendrites, improving the stability and electrochemical performance of solid-state lithium batteries. Recently, Zhang et al. took advantage of this feature of MOFs to develop a high-capacity all-solid-state lithium battery whose anode was fabricated by MOF-based material named Si@MOFs. The experimental results show that the LiFePO_4_//PEO/garnet composite electrolyte//Si@MOF lithium metal solid-state battery maintains 73.1% capacity after 500 cycles at 0.5 C, 60 °C [30]. Similarly, Wang et al. developed an anode based on MOF materials and confirmed that MOF as an anode for solid-state lithium batteries can avoid lithium dendrites and electrode volume changes during cycling. It is found that the 3D interconnected hierarchical structure of the MOF-based material anode provides sufficient nucleation sites for Li, thereby promoting uniform Li plating/stripping and avoiding the generation of dendrites. In addition, the 3D frame structure expands the internal space and effectively buffers the influence of electrode volume changes during cycling. The solid-state lithium battery assembled with the composite electrolyte exhibits excellent cycling and rate performance at 25 °C. This work once again demonstrates that MOF-based materials can be used as dendrite-free lithium metal anodes for solid-state lithium batteries [165]. MOFs can also be used as electrode materials for other solid-state electrochemical devices such as solid-state lithium-oxygen batteries or supercapacitors [166,167].

Since the research on the application of porous polymers in Solid-State lithium battery electrodes is still in its infancy, COFs and MOFs are the first porous polymer materials to be used in electrodes and have achieved promising results, while some reported porous polymer materials have been applied in Lithium-Ion Battery electrodes such as hyper-crosslinked polymers (HCPs), polymers of intrinsic microporosity (PIMs), conjugated microporous polymers (CMPs), and porous aromatic frameworks (PAFs), etc. [168], will be expected to be developed in solid-state lithium batteries.

### 4.3. Summary

Composite polymer electrode materials overcome the disadvantages of high cost, low specific capacity, and low compatibility with electrolytes of traditional transition metal oxides such as LiFePO_4_, LiCoO_2_, LiMn_2_O_4_, etc. However, the ability to resist lithium dendrites is not as good as that of porous polymer materials. Its open porous network structure is beneficial to shorten the ion transport path, and the high specific surface area provides more surface reaction active sites, which promotes the uniform plating/stripping of Li. The generation of dendrites is effectively avoided, and the electrochemical stability is excellent. However, the preparation cost of porous polymers is high, and the types of researches currently invested are limited. It is expected that more porous polymers will be applied in solid-state lithium battery electrodes to promote the development of solid-state lithium batteries.

## 5. Conclusions and Outlook

In summary, the development of solid-state rechargeable Lithium Batteries is facing many challenges at present and the application as well as contribution of polymers in solid-state rechargeable lithium batteries have been reviewed and discussed.

In this paper, solid-state electrolytes including polymer solid-state electrolytes, polymer-inorganic composite solid-state electrolytes, new porous polymer solid-state electrolytes, and electrode materials for solid-state lithium batteries including composite polymers and porous polymer materials are introduced in detail. How polymer materials can improve the conductivity, mechanical properties, compatibility and contact between electrodes and electrolytes of solid electrolytes are also described.

Through comparison, it is found that porous polymers have advantages in the field of solid-state lithium batteries, which are reflected in: (1) high stability; (2) fast lithium-ion transport; (3) ability to resist lithium dendrites, strong mechanical properties; (4) good interface compatibility; (5) Withstand higher current density and voltage. There are some shortcomings that need to be overcome urgently, such as reducing the preparation cost, increasing the yield and diversification of porous polymers, etc. The purpose of summarizing the recent research progress of polymer materials for solid-state rechargeable lithium batteries is to propose conjectures on the future development direction based on the current research level, which is of great significance for the development of new polymer solid-state lithium battery materials.

## Figures and Tables

**Table 2:** Summary of the major performances of MOF-based SSEs.

Solid-State Electrolyte Materials	Ionic Conductivity (S cm^−1^)	Li^+^ Transference Number	Electrochemical Stability Window (V)	Condition	Battery Performance (mAh g^−1^)	Ref.
Li-ILs @ HPCN	1.91 × 10^−4^	0.5	5.2	0.2 C	152.9	[131]
				0.5 C	140.0	
Cu-MOF-74	5.5 × 10 ^−5^	0.36	4.8	0.5 C	152	[128]
PEO-MOF-2	5.20 × 10 ^−4^	0.36	5.0	1.0 C	149.92	[132]
SIPCE-MOF	1.14 × 10^−5^ (30 °C)	0.80	5.68	2 C	105	[127]
	2.23 × 10^−5^ (60 °C)					
HKUST-1@IL-Li	0.68 × 10^−4^ (25 °C)	0.46 (25 °C)		0.5 C	144	[133]
	6.85 × 10^−4^ (100 °C)	0.68 (100 °C)				
UiO-66-NH_2_@SiO_2_	10^−5^ (70 °C)	0.68	3.45	0.1 C	151 (60 °C)	[134]
M-UIO-66-NH_2_/PEGDA (1:8)	4.31 × 10^−5^ (30 °C)		5.5	1 C	80 (30 °C)	[135]
				2 C	110 (60 °C)	
MOF-688	3.4 × 10^−4^ (20 °C)	0.87		30 mA g^−1^	149	[136]
P@CMOF	6.3 × 10^−4^ (60 °C)	0.72	4.97	1 C	107	[137]
ZIF-67 (PLM-2)	1.40 × 10^−6^ (25 °C)	0.41	5.3	0.2 C	130	[138]

**Table 3:** Summary of the major performances of COF-based SSEs.

Solid-State Electrolyte Materials	Ionic Conductivity (S cm^−1^)	Li^+^ Transference Number	Electrochemical Stability Window (V)	Condition	Battery Performance (mAh g^−1^)	Ref.
L@K/C	1.62 × 10^−4^ (30 °C)	0.32	4.2	0.2 C	129	[142]
	4.6 × 10^−4^ (70 °C)					
COF-PVEC	1.11 × 10^−4^ (40 °C)	0.6	4.6	1 C	102.8	[143]
3D-SpCOF	6.4 × 10^−4^	0.7	4.5	0.5 C	141	[144]
Im-COF-TFSI	2.92 × 10^−5^ (30 °C)	0.62 ± 0.02	4.2	0.1 C	123.3	[145]
	4.64 × 10^−4^ (80 °C)					
	4.04 × 10^−3^ (150 °C)					
dCOF-ImTFSI-60@Li	7.05 × 10^−3^ (150 °C)	0.72 ± 0.02	5.32	0.1 C	143.7	[141]
TpPa-SO_3_Li	2.7 × 10^−5^ (RT)	0.9	4.0			[146]
CF3-Li-ImCOF	7.2 × 10^−3^ (RT)	0.81	4.5			[147]
COF-PEO-9-Li	1.33 × 10^−3^ (200 °C)		5.2	3.0 mAg^−1^	120 (100 °C)	[140]
PVDF/H-COF-1@10	2.5 × 10^−4^	0.71	4.3	1 C	128	[148]
